# A dynamic model of annual foliage growth and carbon uptake in trees

**DOI:** 10.1098/rsif.2009.0010

**Published:** 2009-03-11

**Authors:** A. C. Fowler, Oliver Clary, Tiina Roose

**Affiliations:** 1 MACSI, University of Limerick, Limerick, Republic of Ireland; 2 Mathematical Institute, University of Oxford, 24–29 St Giles', Oxford OX1 3LB, UK

**Keywords:** tree physiology, foliage and carbon dynamics, whole-plant model

## Abstract

The growth of trees and other plants occurs through the interactive combination of photosynthesis and carbon (and other nutrient) assimilation. Photosynthesis enables the production of carbohydrate that can then be used in growing foliage, whereby photosynthesis is enabled. We construct a mathematical model of carbon uptake and storage, which allows the prediction of the growth dynamics of trees. We find that the simplest model allows uncontrolled foliage production through the positive feedback outlined above, but that leaf shading provides an automatic saturation to carbon assimilation, and hence to foliage production. The model explains the necessity for finite leaf area production at outbreak, and it explains why foliage density reaches a constant value during a growing season, while also non-leaf tissue also continues to grow. It also explains why trees will die when their carbon stores are depleted below a certain threshold, because the cost of foliage growth and maintenance exceeds the dynamic supply of carbon by photosynthesis.

## 1. Introduction

In the current ongoing debate about climate change, the role of the carbon cycle is of major importance (Bigg 2003). The net flux of carbon (as CO_2_) to the atmosphere is some 2 Gt (gigatonnes or 10^12^ kg), but the contribution from fossil fuel consumption is more than twice this, and deforestation is almost as important as that. In fact, the fluxes between the various reservoirs are much larger, and so an understanding of the way in which carbon is transferred is of some importance.

The growth of plants and trees is an interesting and important process in the discussion of climate, because of their huge importance as a sink of CO_2_ in the atmosphere: the net removal by vegetation is some 5 Gt a year. Therefore the quantitative and predictive understanding of carbon uptake by plants is an issue of much current interest. On the other hand, the consequent effect of climate change on the dynamics of plant growth is of equal importance for the understanding of crop dynamics.

The interaction of plant growth with carbon uptake is a problem in plant physiology, which has attracted much attention in the botanical and ecological literature, but less so in the applied mathematical community. An understanding of the processes of development and growth is important both scientifically and practically, since the response of plants and trees to nutrient deficiency and attacks by viruses and pests has important implications for crop and forestry management. In this paper, we develop and analyse a mathematical model for the growth of trees, which considers the continuous evolution of two critical variables, the carbon store and the foliage density. Our model finds its origins in the pioneering work of Thornley (1976; see also Thornley & Johnson 1990), and indeed our model closely resembles the whole plant model developed in Thornley's book (see his eqns (7.13) and (7.14)). However, we go beyond Thornley's model in two respects. The first is that we give a derivation of the equations of the model from first principles, describing the processes of water and nutrient transport within the tree; and second, we analyse the model and show that, in its simplest form, it exhibits an unphysical indefinite growth of foliage. We then go on to include the effects of leaf shading and show that the resultant augmented model allows for growth to a stable steady state, consistent with annual foliage development in mature trees. In a subsequent paper, we will use this model as a constituent in studying the defoliative effects on mountain birch forests in Lapland of outbreaks of the autumnal moth *Epirrita autumnata*, and with this in mind we will parametrize the present model with values suitable for the growth of a mountain birch.

The outline of the paper is the following. In §2 we describe the necessary tree physiology to develop the model, and in §3 we develop the model from first principles. We show that the model allows unbounded growth, and thus in §4 we seek a modification that prevents this happening. This is at hand in the form of leaf shading (Thornley 1976, ch. 3), which causes the photosynthetic rate to become saturable. We show in §4 that the inclusion of this effect causes the model to behave in ways consistent with observation. Conclusions follow in §5.

## 2. Tree physiology

We begin by discussing the physiology of tree growth, with specific reference where appropriate to mountain birch. Early growth of foliage in trees is thought to depend on carbohydrates accumulated during the previous growing season. Carbohydrates are the direct product of photosynthesis, and are involved in many different processes in plants, such as respiration and maintenance. The leaves use carbon dioxide, water and energy from sunlight to produce carbohydrates, which are then translocated to different parts of the tree, which need them. A significant fraction of carbohydrate produced from photosynthesis is used for the growth of plant tissue (new wood in the case of trees). Another large fraction is stored for use during times when there is no production from photosynthesis, and a large amount of this stored carbohydrate is then used for initial leaf growth in early spring. There is also a considerable amount of carbohydrate that is oxidized in respiration, which releases the energy for tree growth and metabolic processes.

### 2.1. Vascular system

Nearly all living plants have a vascular system that is crucial for the transport of water, minerals, food and other substances to the different parts of the plant. This vascular system consists of two main pathways: the xylem and the phloem. The main difference between these is that the phloem is used to transport substances down the plant, whereas the xylem is used to transport substances (water and other minerals) up the plant. The phloem is considered to be the main pathway for carbohydrate transport, and generally very little carbohydrate is thought to travel in the xylem.

It is thought that most of the reserve carbohydrates are stored in parenchyma cells in the xylem of the trunk for birch species. This was found by Olofinboba (1969) while comparing storage carbohydrates in African and European trees. These storage cells are thought to constitute 10 per cent of the xylem; the rest of the pathway is made up of dead cells, i.e. their only function is structural, and they do not store carbohydrate. Xylem is the main pathway for the transport of water and nutrients up the tree; the phloem is the main pathway for the products of photosynthesis down the tree. Transport down the phloem is driven largely by osmotic pressure differences; the flow of water up the xylem is driven by a pressure gradient that is induced by transpiration at the leaves, such that the water pressure in the xylem is below the saturation vapour pressure. This is known as the cohesion–tension theory (Dixon & Joly 1895) and, although well accepted, it is still controversial (Tyree 1997).

Xylem is made up of microscopic tubular vessels, which are distributed across the trunk in small clusters. Takahashi (1987) measured the diameters and distributions of xylem vessels in six birch species that grow in Siberia. For a birch species with characteristics similar to those of mountain birch, he measured an average vessel diameter of 47 μm, and a distribution of approximately 100 per mm^2^, with an average spacing of 23 μm. The vessels are never much longer than 4 mm, but the transported water can easily pass from one vessel to another by passing through small pits before entering another xylem vessel. For simplicity, we will suppose the vessels run continuously up the tree trunk.

Mineral nutrients and carbohydrates are transported in the vascular system as solutes in water. We suppose that stored carbohydrate in parenchyma cells is released to the water in the system in a diffusive fashion.

### 2.2. Photosynthesis

The production of carbohydrates occurs through photosynthesis in the leaves of the plant. Photosynthesis is the mechanism that reduces atmospheric CO_2_ to carbohydrates using the energy from light and the release of oxygen from water. The basic chemical reaction can be described by2.1
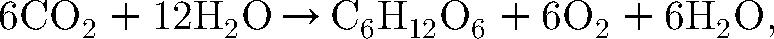

and occurs at chloroplast cells within the leaves. These lie below the epidermis of the leaves, in which there are small openings called stomata. The stomata open and close, depending on climatic conditions such as temperature and wind strength. Atmospheric CO_2_ is absorbed through the stomata, where it reacts as in (2.1) with water derived through the xylem from the roots. The excess water produced is then released by evaporation to the atmosphere through the stomata, this process being called transpiration, while the products of photosynthesis, the photosynthates, are transported away from the leaf through the phloem.

The carbohydrates thus produced are removed to the parts of the tree where they are needed, and to the storage cells. The amount of carbohydrate produced depends on how much water, CO_2_ and light are available, and on how much foliage is present. Many experiments have been performed to measure how much CO_2_ is absorbed in plants and then transformed into carbohydrate, and the results can vary considerably between species (James 1973; Kozlowski & Pallardy 1997; Newell *et al.* 2002).

## 3. A mathematical model

There are three different types of carbohydrates (monosaccharides, oligosaccharides and polysaccharides) present in most plants (Barbaroux *et al.* 2003), but we will lump these together in our model. One of the variables of the model will thus be the carbon store, denoted C, which is measured in units of weight of carbohydrate, kilograms of carbohydrate (kgC). A second variable is the amount of foliage, denoted F, which is measured in units of weight of foliage, kilograms of foliage (kgF). The final variable is the woody biomass, denoted W, which is measured in units of weight of woody biomass, kilograms of woody biomass (kgW). More realistic models would include further compartments, but we avoid such complications here for purposes of simplicity.

We model the continuous variation of foliage, woody biomass and carbon by three ordinary differential equations, which take the form3.1
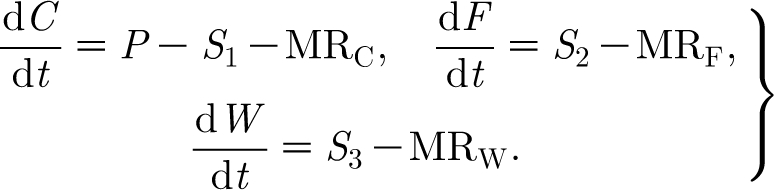

In these equations, *P* denotes the rate of production of carbohydrate by photosynthesis; *S*_1_ denotes the rate of consumption of carbohydrate in foliage and woody tissue production; MR_C_ denotes the rate of consumption of carbohydrate in supporting metabolic processes; *S*_2_ is the rate of production of foliage; MR_F_ represents foliage respiration; *S*_3_ is the rate of production of woody biomass; and MR_W_ is the rate of woody biomass respiration.

The production of carbohydrate by photosynthesis is assumed to be proportional to leaf surface area, and we therefore take it to be proportional to *F*, thus3.2
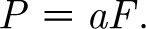

Thornley (1976) suggested a similar relationship. The coefficient *a* will depend on received photosynthetically active radiation and water supply. A discussion of this (assuming wet conditions) is given by Thornley & Johnson (1990, p. 224 ff.). In the present notation, we write3.3
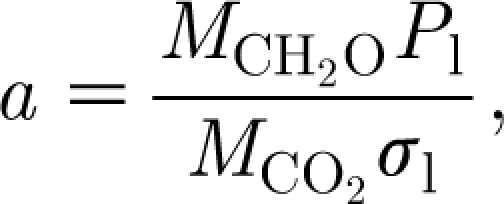

where *σ*_l_ is the leaf areal density, in units of kgF m^−2^, and *P*_l_ (Thornley & Johnson's *P*_n_) is the specific photosynthetic rate, in units of kgCO_2_ m^−2^ d^−1^. The molecular weights 
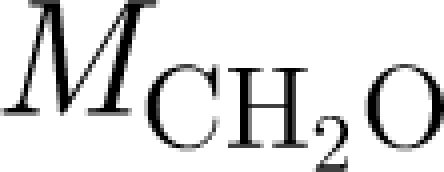
 of organic carbon and 
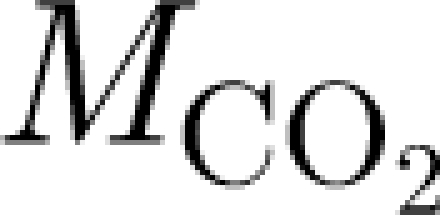
 of CO_2_ provide the relevant conversion from carbon dioxide weight to carbohydrate weight. *P*_l_ is described by a diffusive flux from the atmosphere, thus3.4
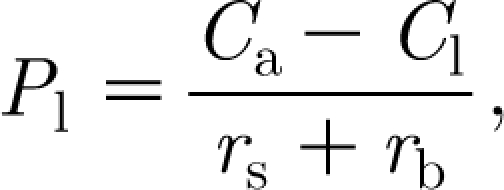

where *r*_s_ and *r*_b_ are stomatal and boundary-layer resistances, respectively, measured in units of d m^−1^, and *C*_a_ and *C*_l_ are free air and leaf air concentrations of CO_2_, in units of kg m^−3^. Thornley & Johnson suppose a specific leaf respiration rate of *R*_d_, which is the local production rate of CO_2_. The leaf CO_2_ concentration is then determined by equating the net production of CO_2_ to its removal by photosynthesis, described by a Michaelis–Menten-like pair of equations; this leads to3.5
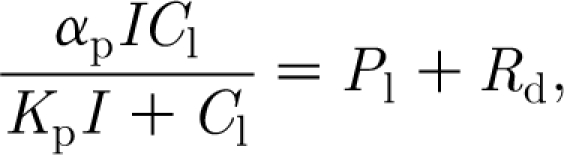

where *I* is the leaf irradiance, and *α*_p_ and *K*_p_ are constants associated with the photosynthetic reactions. From these, we can eliminate *C*_l_ (as the unique positive solution of a quadratic equation), and thus obtain an expression for *a* in terms of *I*, *C*_a_ and *r*_s_+*r*_b_. In the following, we assume it to be constant, as is commonly done (e.g. Magnani *et al.* 2000).

The metabolic rates MR_C_, MR_F_ and MR_W_ are simply taken to be proportional to *C*, *F* and *W*, respectively, thus3.6
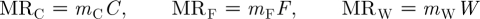

and then the specific leaf respiration rate *R*_d_ is given by3.7
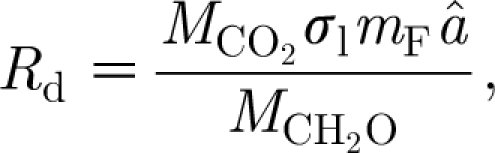

where 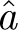 (kgC kgF^−1^) is the carbohydrate fraction of leaf tissue. It is clear that the rates in (3.6) must go to zero as *C*, *F* and *W* go to zero, respectively. Since MR_F_ represents a decay rate, a linear dependence seems appropriate. Carbon use must saturate at high levels of carbon, since the tree has a finite capacity for carbon uptake. We ignore this here, partly because our later concern will be with trees under attack, where it is more likely that a linear dependence of metabolism on *C* is appropriate.

The sink and source terms *S*_1_, *S*_2_ and *S*_3_ are more complicated. If *S*_1_ is known, then we assume that3.8
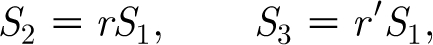

where *r* and *r*′ are conversion factors that determine foliage and woody biomass production in terms of carbohydrate consumed. These are simplifying assumptions, since they suppose a direct connection between foliage production and carbon release from storage. Thornley (1976) proposes a similar connection between the sink and source terms.

It then remains to prescribe *S*_1_, and we discuss this in some detail. First, we find in appendix A that the water flux *q* is defined implicitly by the equation3.9
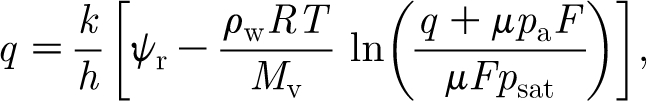

where3.10
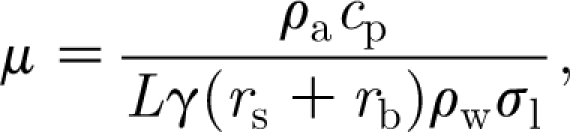

and the various constants are defined in appendix A, as well as in table 1.

**Table 1:** Assumed typical values of the constants of the model. (Units: 1 kPa=10^3^ Pa; 1 day=8.64×10^4^ s.)

symbol	value	meaning
*a*	0.08–0.1 kgC kgF^−1^ d^−1^	photosynthetic assimilation rate; Hoogesteger & Karlsson (1992) and Sveinbjörnsson (2001)
*A*_c_	3 m^2^	tree canopy area; Aradottir *et al.* (2001)
*c*_p_	10^3^ J kg^−1^ K^−1^	specific heat of moist air; Tan *et al.* (1978)
*d*	0.6×10^−4^ m	xylem vessel diameter; Petty (1978)
*D*	0.37×10^−11^ m^2^ s^−1^	carbohydrate diffusivity; Briggs & Robertson (1948, p. 276); Steward (1930), tables 3 and 4
*h*	2 m	tree height; Aradottir *et al.* (2001)
*k*_l_	0.6	leaf shading parameter; McMurtrie & Wolf (1983)
*l*	1.2×10^−4^ m	xylem vessel spacing; Petty (1978)
*L*	2.45×10^6^ J kg^−1^	latent heat; Tan *et al.* (1978)
*m*_C_	0.5×10^−3^ d^−1^	carbon metabolic rate; Kozlowski & Pallardy (1997, p. 139)
*m*_F_	0.015 d^−1^	foliage metabolic rate; Kozlowski & Pallardy (1997, pp. 16, 146); Thornley & Johnson (1990, p. 265)
*m*_W_	0.5×10^−3^ d^−1^	woody biomass metabolic rate; Kozlowski & Pallardy (1997, p. 139)
*M*_v_	18×10^−3^ kg mol^−1^	molecular weight of water
*p*_a_	=*Hp*_sat_, e.g. 1.4 kPa	air water vapour pressure
*p*_sat_	1.7 kPa	saturation water vapour pressure (at 15°C)
*P*_0_	5 kgC m^−2^ yr^−1^	photosynthetic rate; McMurtrie & Wolf (1983)
*r*	0.2 kgF kgC^−1^	foliage conversion factor; Barbaroux *et al.* (2003)
*r*′	0.3 kgF kgC^−1^	woody biomass conversion factor; Barbaroux *et al.* (2003)
*r*_b_	<3 s cm^−1^	boundary-layer resistance; Tan *et al.* (1978) and Kozlowski & Pallardy (1997)
*r*_s_	1–50 s cm^−1^	stomatal resistance; Tan *et al.* (1978) and Kozlowski & Pallardy (1997)
*R*	8.3 J mol^−1^ K^−1^	universal gas constant
*T*	288 K	ambient absolute temperature
*V*_s_	0.006 m^3^	carbon volume store (we use 10% of an estimated tree volume of 0.06 m^3^)
*w*	5×10^−6^ m	xylem vessel wall thickness; Kukkola *et al.* (2004)
γ	0.066 kPa K^−1^	psychrometric constant; Tan *et al.* (1978) and Ventura *et al.* (1999)
*η*	1.1×10^−3^ Pa s	viscosity of water; Batchelor (1967)
*ρ*_a_	1.2 kg m^−3^	moist air density; Tan *et al.* (1978)
*ρ*_c_	300 kgC m^−3^	carbohydrate concentration in wood; Barbaroux *et al.* (2003)
*ρ*_w_	10^3^ kg m^−3^	water density; Batchelor (1967)
*σ*	0.1–0.2	available carbohydrate fraction; Barbaroux *et al.* (2003)
*σ*_l_	0.05–0.08 kgF m^−2^	leaf areal density; Hoogesteger & Karlsson (1992); Sveinbjörnsson (2001)
*ψ*_r_	−0.2 MPa	root hydraulic potential; Tyree & Ewers (1991)

This defines *q* implicitly in terms of *F*. Using values from table 1, we find *p*_w_≈133 MPa, which compares with the typical measured leaf values of hydraulic potential of *ψ*_l_≈−0.7 MPa. Because we expect 
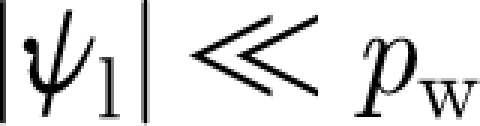
, it follows from (A 5) that *p*_l_≈*p*_sat_, which thus defines *q* through (A 4) and (A 3), and then (A 1) determines the value of *ψ*_l_, given *ψ*_r_. Specifically, we have3.11
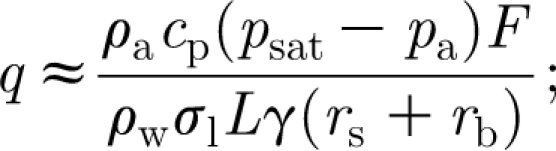

note that this is independent of tree height *h*.

To calculate the carbon transport to the foliage, we denote by *c*_w_ the concentration of carbon in the water in the xylem, measured as the weight of carbohydrate equivalent per unit volume of water (kgC m^−3^), so that *S*_1_=*qc*_w_ is the flux to the leaves. In appendix B, we show that3.12
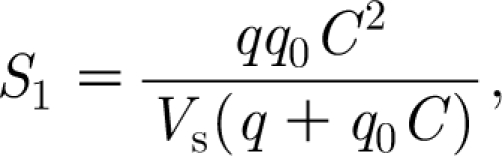

where3.13
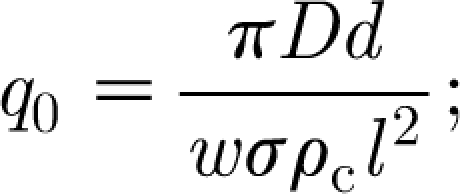

the new constants are defined in appendix B and table 1.

Gathering together (3.2), (3.6), (3.8) and (3.12), equation (3.1) can be written in the form3.14
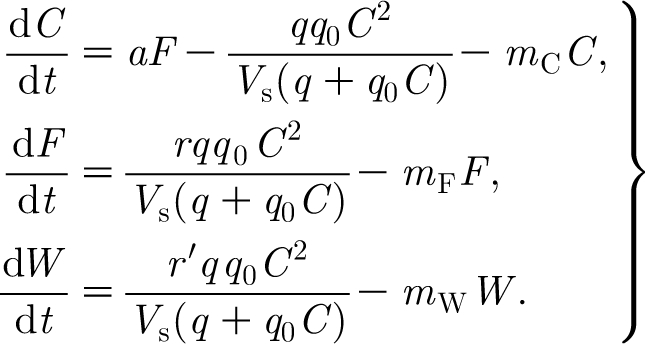

The flux *q*(*F*) is implicitly defined by (3.9) and, we anticipate, by (3.11).

### 3.1. Non-dimensionalization

To simplify the model, we scale the variables as3.15
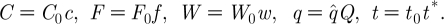

We define the relative humidity3.16
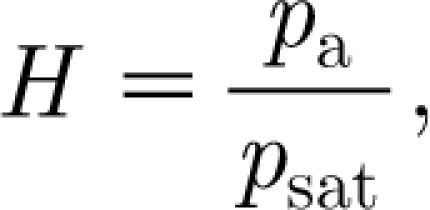

and choose3.17
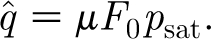

We anticipate that 
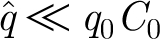
, and an appropriate balance of terms then suggests that we choose3.18
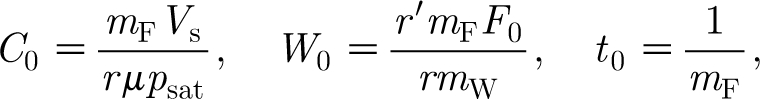

with *F*_0_ being as yet undetermined. The corresponding dimensionless equations are then3.19
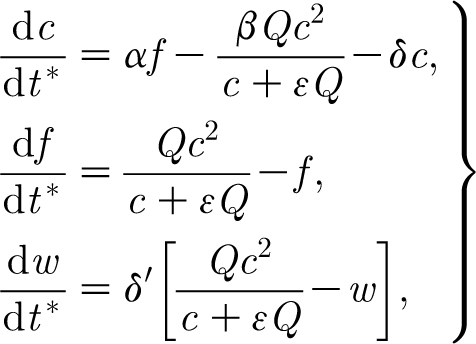

where the dimensionless parameters are defined by3.20
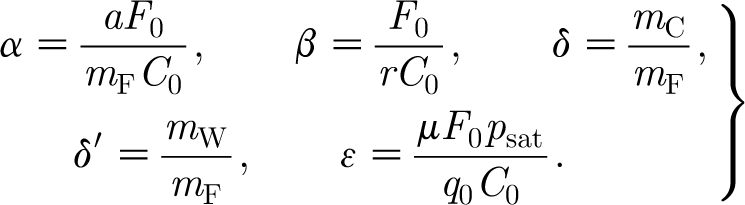

The dimensionless version of (3.9) is then3.21
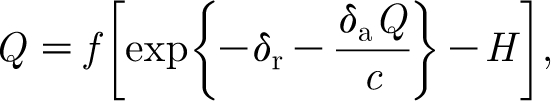

where3.22
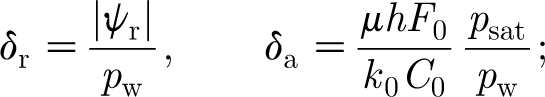

*p*_w_ is defined in (A 5) and *k*_0_ in (B 8).

To estimate the scales and parameters, we use the values in table 1. These values have been chosen for mountain birch where possible, or, where not, from trees of similar size and from similar climates. Using these values (and particularly *σ*_l_=0.08 kgF m^−2^, *r*_s_+*r*_b_=40 s cm^−1^, *σ*=0.1, *a*=0.1 kgC kgF^−1^ d^−1^), we find3.23
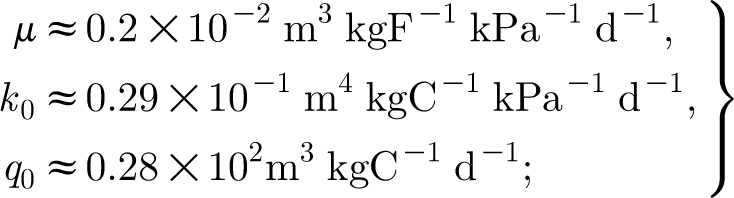

then we find the scales3.24
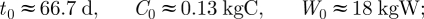

if we take a nominal value of *F*_0_=0.4 kgF, then we obtain the dimensionless parameters3.25
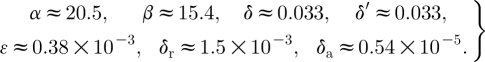



### 3.2. Phase plane analysis

A first comment concerns the woody biomass equation for *w*. Because 
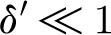
, we see that *w* changes little over the course of a growing season and can be taken as approximately constant. This of course simply represents the fact that trees grow over a much longer time scale than a year. In any case, the equation for *w* uncouples from those for *c* and *f*, unless account is taken of the variation of stomatal conductance 1/*r*_s_, and thus *μ*, and thus *α* and *β* (via *C*_0_), on tree height (and hence *w*) (Ryan *et al.* 2000). This dependence would cause *α* and *β* to decrease somewhat with *w*, but, since *w* itself changes slowly, this coupling has little effect on a seasonal time scale. We thus formally put *δ*′=0 and consider only the first two equations in (3.19).

We begin by ignoring the very small terms in *ϵ*, *δ*_r_ and *δ*_a_. We then have3.26
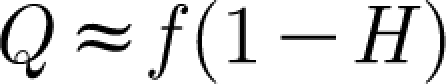

(which corresponds to (3.11)), and the equations (3.19) take the approximate form3.27
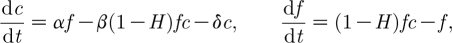

where, for convenience, we now drop the asterisk on the dimensionless time *t* ^*^.

These two ordinary differential equations are easily studied in the (*c*,*f*) phase plane. The solutions should represent the evolution of carbon store and foliage density during a growing season. We might expect that *f* would increase towards a steady state, and carbon store might do so as well or gradually increase. Carbon stores are depleted during winter when photosynthesis is inoperative.

**Figure 1:**
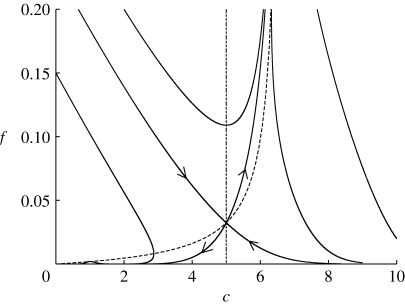
Phase portrait for (3.27) in the vicinity of the unstable fixed point at *c*≈1/(1−*H*), *f*≈*δ*/((1−*H*)(*α*−*β*)), using values *α*=20.5, *β*=15.4, *δ*=0.033 and *H*=0.8. The dashed lines are the nullclines.

There are two steady states, *c*=*f*=0, and3.28
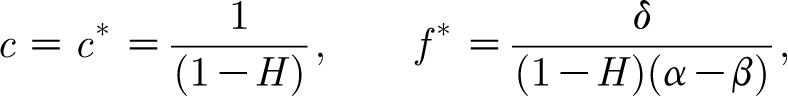

which is in the positive quadrant if, as we assume, *α*>*β* (and the relative humidity *H*<1). On *c*=0, 
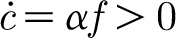
, while the *c*-axis is invariant. Thus, if *f* and *c* are initially positive, they remain so. The nullclines are given in the positive quadrant by 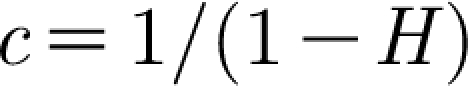 and *f*=0 for 
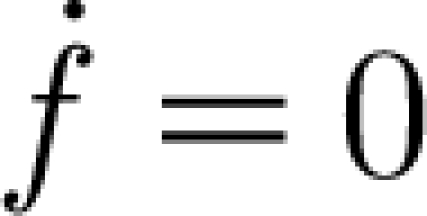
, and 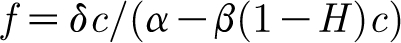 for 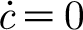. These intersect at (*c* ^*^,*f* ^*^), which is a saddle point. Trajectories lying below the two stable separatrices that approach (*c* ^*^,*f* ^*^) tend towards the origin, which is a stable node, while those above asymptote towards the upper unstable separatrix from (*c* ^*^,*f* ^*^), and both *c* and *f* grow unboundedly. The phase portrait is shown in figure 1.

The phase plane in figure 1 has a good point as well as a bad one. It suggests that if the tree is initially depleted of carbon 
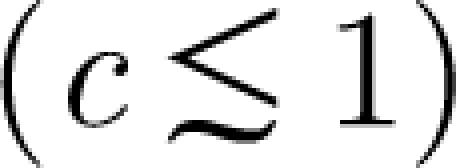
, then, if there is insufficient initial foliage, the carbon store will be depleted and the foliage will die: the tree will not survive. If 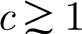, then the same fate can occur, but only if the initial foliage is very small. Note that the initial foliage must be positive for the tree to survive; this is consistent with the fact that, at budbreak, the tree does indeed produce an initial positive foliage density, depleting its carbon store to do so, as indicated by the trajectories in 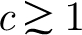. However, the model then predicts unbounded growth in both carbon and foliage through an uncontrolled positive feedback between the two. It is this unwanted behaviour that we seek to redress in §4.

## 4. Leaf shading

One simple way in which we might suppose that foliage growth is curtailed is through the same mechanism that limits the growth of trees themselves. Although other suggestions have been made, Ryan & Yoder (1997) plausibly suggested that tree growth becomes limited in association with the height of the tree *h*. The mechanism of this hydraulic limitation hypothesis is that, as *h* increases, the tension in the water column in the xylem must increase (*ψ*_l_ decreases in (A 1)) to drive the same water flow, making the xylem more prone to cavitation and thus damage. To prevent this, leaf stomatal conductance (1/*r*_s_) is reduced, and this decreases photosynthetic rate and thus also the carbon available for woody (and perhaps foliage) growth. The hypothesis has been supported by Schäfer *et al.* (2000), Delzon *et al.* (2004) and Addington *et al.* (2006), for example, although not conclusively, and others do not agree (e.g. Becker *et al.* 2000). In the present model, hydraulic limitation does not seem a likely cause for reducing foliage growth, since our description is essentially appropriate for mature trees, and because there is actually no serious dynamic effect of reducing water flow in the model (3.19): reduced stomatal conductance 1/*r*_s_ causes reduced *μ* in (3.10), and thus increased *C*_0_ in (3.18). From (3.20), this reduces *α* and *β* proportionally, so that the critical inequality *α*>*β* is unaltered. The phase plane of figure 1 is unaltered, and the problem of unbounded foliage growth remains. We may note that the choice of respiratory terms in (3.14) is actually consistent with the respiration hypothesis (Ryan & Yoder 1997), although we should also emphasize that the issue of tree height is not central to our thesis.

There are two covert assumptions in the model equation (3.1), which we might alter in seeking a resolution to the prediction of uncontrolled foliage growth. One was mentioned after (3.6); that is, the carbohydrate metabolization term *M*_C_ must saturate at large *C*. Since in the dimensionless model this term is small, we do not expect that its modification at a large *c* will have any serious effect on the solution behaviour.

The other assumption is that the rate of photosynthesis is proportional to leaf area and thus foliage density. This assumption ignores the fact that sunlight reaching the surface of a leaf will depend on the degree to which it lies in shade, and leaves deeper within the canopy will receive less sunlight. This leads to the assumption that photosynthesis is a saturable function of foliage *F*, such as described by McMurtrie & Wolf (1983) and West (1987) who supposed that *P* in (3.1) was given by4.1
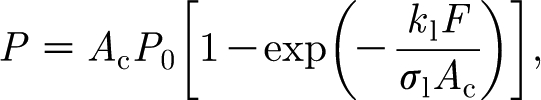

where *σ*_l_ is the leaf areal density introduced before; *A*_c_ is the tree canopy area (assumed constant); and *k*_l_ is a number, taken illustratively as 0.6 by McMurtrie & Wolf (1983). Introduction of (4.1) into the dimensionless model yields the corresponding dimensionless equations (in the approximated form analogous to (3.27))4.2
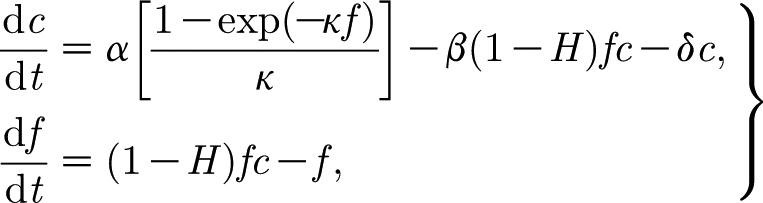

where *α* retains its definition in (3.20) provided we define4.3
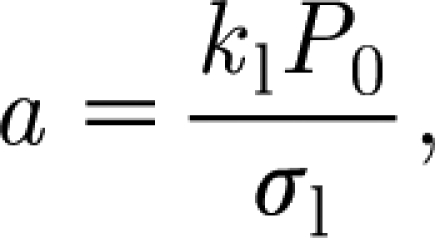

and the parameter *κ* is given by4.4
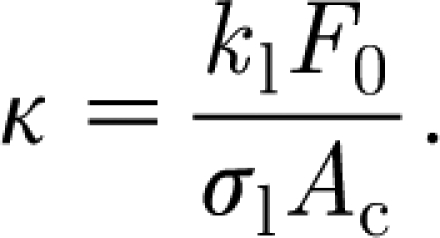

With the values in table 1, we find that *a*=0.1 (as estimated independently) for the value *σ*_l_=0.08 kgF m^−2^.

Since, in fact, we hope that leaf shading will provide a limiting mechanism for carbon and foliage growth, it is natural to assume that *κ*∼*O*(1), and we can finally use this to *choose F*_0_ so that *κ*=1, i.e.4.5
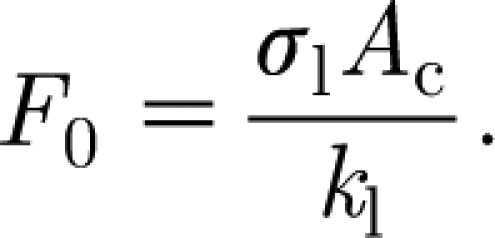

With the values in table 1, we find *F*_0_=0.4 kgF, as previously assumed, and therefore the values of *α*, *β* and *δ* retain their previous estimated values.

Taking *κ*=1, the model (4.2) takes the form4.6
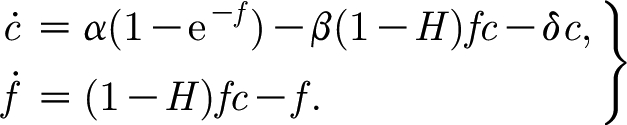



This has a fixed point as before where *f*∼*δ*, and the local structure of the phase plane is the same as in figure 1. In addition, there is a further fixed point where *c*=*c*_0_=1/(1−*H*), and *f*=*f*_0_=*O*(1) is the larger root of the equation4.7
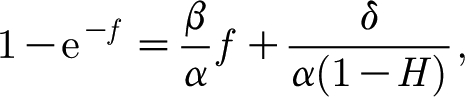

which has such an *O*(1) solution for 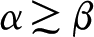.

The fact that *α* and *β* are large (and 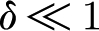) enables us to describe the dynamics of the solution when *f*=*O*(1). First, *c* rapidly approaches a quasi-steady state given by4.8
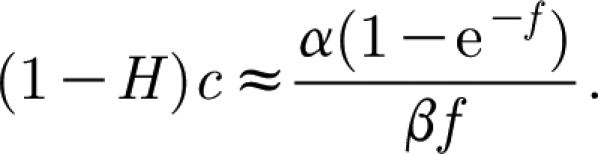

The right-hand side of this expression is a monotonically decreasing function of *f*, which equals (*α*/*β*)>1 when *f*=0 and tends to zero as *f*→∞. The migration of *f* along this curve is given by4.9
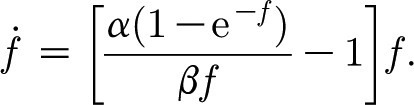

**Figure 2:**
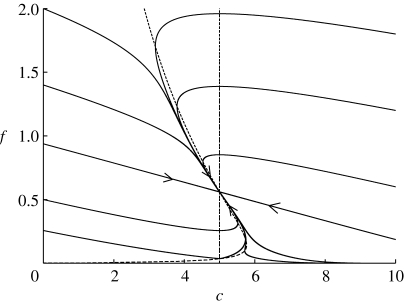
Phase portrait for (4.6) using values *α*=20.5, *β*=15.4, *δ*=0.033 and *H*=0.8. The lowest curve on the left, which leaves the *f*-axis at (0, 0.257), is almost the separatrix to the unstable saddle at *c*=5, *f*≈0.032, which can be approximately seen by the kink in this trajectory. The dashed lines are the nullclines.

The right-hand side of (4.9) is a unimodal (one-humped) function with a unique positive steady state at *f*=*f*_0_ (the positive solution of (4.7)) which is globally stable. From this we understand that the steady state (*c*_0_,*f*_0_) is a stable node. The phase portrait for the system is shown in figure 2. The dynamics for small *f* are still described by figure 1, which is thus a close-up of figure 2 near the unsteady saddle (not readily discernible in figure 2).

## 5. Conclusions

In this paper, we have presented a relatively simple model, similar to others in the literature, in which we describe the dynamics of carbon assimilation, storage and use in mature trees, with a view to describing how the carbon storage evolves over the growing season. We have focused our attention on the mountain birch, because in a succeeding paper we will describe how this dynamic evolution is affected by the presence of the larvae of the moth *E. autumnata*. Our model presents two advances over previous work. First, the dynamics of carbon uptake and transport within the xylem is described physically, so that the nonlinearity of the model is based on coherent physical principles. Second, we study the global dynamic behaviour of the model in a way that has not been done before. In particular, we find that the model provides a descriptive reason for why at budbreak leaves form with a small but finite capacity for photosynthesis. In addition, we show that leaf shading provides a practical mechanism by which foliage growth is limited and the carbon storage reaches a steady state.

Both of these features provide insight into the mechanisms of carbon regulation during tree growth. The stable steady state shown in figure 2 at *c*=5, *f*≈0.56 corresponds to a carbon store of 0.65 kgC and a foliage quantity of 0.22 kgF. During the winter the carbon store is depleted, so that in spring the initial values (in dimensionless units) will be *c*<5 and *f* small. For example, the separatrix in figure 2 from *c*=0, *f*=0.257 goes approximately through the values *c*=3, *f*=0.1. So if *c*=3 at the end of winter (corresponding to 0.39 kgC), then for an initial foliage less than *f*=0.1 (corresponding to 0.04 kgF), carbon will first increase, but then irreversibly decrease, and tree death will ensue.

**Figure 3:**
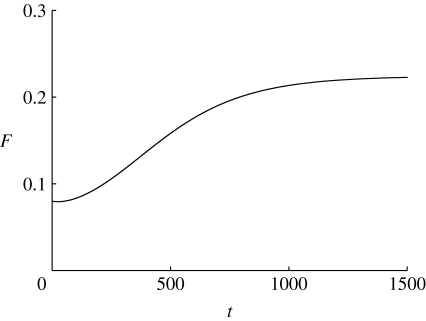
Foliage in kgF as a function of time in days, found by solving (4.6), using parameter values *α*=20.5, *β*=15.4 and *δ*=0.033, and with the choice of scales *F*_0_=0.4 kg, *C*_0_=0.13 kg, *t*_0_=66.7 d and *H*=0.8.

**Figure 4:**
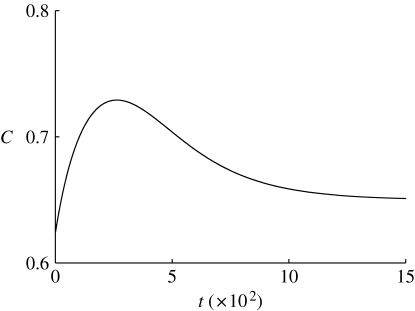
Carbon store in kgC as a function of time in days, using the same parameters as in figure 3. Note that the axis for *C* starts at 0.6.

In reality, we would expect the carbon store to be less depleted. Figures 3 and 4 show the time series of foliage and carbon quantities as a function of time (in dimensional units) starting from dimensionless values *c*=4.8, *f*=0.2, corresponding to *C*=0.62 kgC and *F*=0.08 kgF. There is an initial slight dip in foliage quantity before it rises monotonically towards its final steady state. The carbon store initially rises as photosynthesis is initiated and then is depleted by the growth of foliage. Depending on the parameters that are chosen, the final approach to the steady state can be somewhat oscillatory, which is probably unphysical.

Figures 3 and 4 do highlight a shortcoming of this model. Although we have attempted to use the best estimates available for the parameters, we see that the time scale for approach to equilibrium is much too long, of the order of 1000 days. This is too long by a factor of perhaps 30. The time scale is given by *t*_0_=1/*m*_F_, where *m*_F_ is the specific respiration rate. In this simple model, somewhat analogous to simple first-order rate laws used in epidemiology, we are actually assuming an exponential distribution of foliage lifetime with a mean of 1/*m*_F_=67 days; evidently this is much too long, and the model ought to be refined, perhaps by means of specific inclusion of foliage lifetime as well as foliage respiration.

A simpler alternative is just to shorten the time scale by increasing *m*_F_. There is then a consequent effect on *α*, *β* and *δ*. If one does increase *m*_F_, then indeed the time scale is shortened, but the dynamics becomes increasingly oscillatory, which also seems implausible. We think that this shortcoming represents an issue concerning the veracity of the model which needs to be addressed. For example, specific respiration rates decrease rapidly with foliage age (Kozlowski & Pallardy 1997), and this would have a compensating effect. However, we also think that the robust qualitative conclusions of the present model provide a useful basis on which more elaborate and realistic models can be built.

## Appendix A

In this appendix, we derive an expression for the water flux *q* through the xylem. This is given by

A1

where *h* is the tree height; *ψ*_r_ and *ψ*_l_ are the hydraulic potentials in roots and leaves, respectively; and the hydraulic conductivity *k* is supposed to be given by a formula based on the assumption of Poiseuille flow through *n* xylem vessels of diameter *d*,

A2

where *η* is the viscosity of water (Tyree & Ewers 1991).

The flux of fluid to the leaves is essentially balanced by evaporation from the foliage, and we suppose that this is proportional to the quantity of foliage, thus

A3

where *E* is the evaporation rate measured in kg m^−2^ d^−1^ (1 d=1 day=8.64×10^4^ s), and *ρ*_w_ is the density of water. Tan *et al.* (1978) give an expression for the evaporation rate which can be written in the form

A4

where *p*_l_ and *p*_a_ are the water vapour pressures at the leaf and in the atmosphere, respectively; *ρ*_a_ is the density of moist air; *c*_p_ is the specific heat of moist air; *L* is the latent heat of vaporization of water; and *γ* is the so-called psychrometric constant. The stomatal conductance 1/*r*_s_ is a decreasing function of the vapour pressure deficit *p*_l_−*p*_a_ (Oren *et al.* 1999), so that the evaporation rate *E* in (A 4) grows more slowly than linearly and may saturate at high vapour pressure deficit. The derivation of equation (A 4) is given in appendix C.

In order to proceed, we must relate the hydraulic potential at the leaf, *ψ*_l_, to the water vapour pressure *p*_l_. This is done in appendix D, where we find the relationship

A5

where *p*_sat_ is the saturation vapour pressure; *ρ*_w_ is the density of water; *R* is the universal gas constant; *T* is the absolute temperature; and *M*_v_ is the molecular weight of water.

We eliminate *ψ*_l_ and *p*_l_ from (A 1) and (A 3) using (A 4) and (A 5) to obtain

A6

where

A7

## Appendix B

We assume that the carbon transport to the foliage is equal to the flux from the carbon store. If *V*_s_ is the volume of the carbon store, then *C*/*V*_s_ is the effective concentration of stored carbon. We suppose that stored carbon enters the xylem diffusively, so that the relationship equating the flux to the foliage with that to the xylem (kgC s^−1^) is

B1

where *D* is a diffusion coefficient and *w* is the width of xylem vessel walls. It follows from this that the xylem concentration of carbon is

B2

where

B3

Finally, we allow for the fact that the number of xylem vessels is related to the carbon store, most simply because the carbon is stored in parenchyma cells in the xylem. Put another way, if *ρ*_c_ is the density of structural carbohydrate in the wood, then the total mass of carbon in the trunk is approximately *ρ*_c_ *hA*, where *A* is the trunk cross-sectional area. If the carbon reserves available for growth are a fraction *σ* of this, then

B4

while an estimate for the number of xylem vessels is

B5

where *l* is the xylem spacing. This leads to

B6

From (A 2) and (B 3), we have

B7

where

B8

from these and (B 2), we finally derive an expression for the carbon flux to the leaves,

B9

## Appendix C

The evaporative flux *E* of water vapour across a layer is defined by *E*=Δ*ρ*/*r*, where Δ*ρ* is the water vapour density difference across the layer and *r* is the resistance. For resistances across stomata and a turbulent boundary layer in series given by *r*_s_ and *r*_b_, respectively, we can write

C1

where *ρ*_l_ is the stomatal water vapour density and *ρ*_a_ is that in the free atmosphere. The expression in (A 4) then follows provided we define *γ* to be

C2

where Δ*p*=*p*_l_−*p*_a_, Δ*ρ*=*ρ*_l_−*ρ*_a_.

We use the Clausius–Clapeyron equation and perfect gas law for water vapour in the form

C3

where *M*_v_ is the molecular weight of water vapour; from these, we can derive

C4

and thus the psychrometric constant *γ* can be defined as

C5

## Appendix D

We wish to relate the water vapour pressure in the stomata, *p*_l_, to the hydraulic potential in the leaf, *ψ*_l_. To do this, we note that the chemical potentials of water vapour in the stomata and of water in the leaf are equal, say, to *μ*_l_. In the vapour, we have

D1

where the superscripts denote reference values, while in the leaf

D2

where *V*_m_ is the molar specific volume of water,  being the molecular weight of water and *ρ*_w_ being its density.

We relate the liquid and gas reference chemical potentials by

D3

where *p*_sat_ is the saturation vapour pressure. Putting these together, we find

D4

as given in (A 5).
